# Internal Sodium-Potassium Translocation Drives the Growth Adaptation of *Suaeda salsa* in a Coastal Wetland Ecosystem

**DOI:** 10.3390/plants15111668

**Published:** 2026-05-29

**Authors:** Lichao Zhang, Kaipeng Zhang, Jingyu Yang, Huimin Lou, Xuepeng Liu, Wenjun He, Dongjie Zhang

**Affiliations:** Shandong Key Laboratory of Eco-Environmental Science for the Yellow River Delta, Shandong University of Aeronautics, Binzhou 256600, China; zhanglc_0225@163.com (L.Z.); zkpzk666@163.com (K.Z.); 19709793970@139.com (J.Y.); 17865609609@163.com (H.L.); 17686378490@163.com (X.L.)

**Keywords:** Na-K transport coefficient, growth adaptation, Yellow River Delta wetland, halophyte, Na-K homeostasis

## Abstract

Understanding the internal translocation of sodium (Na) and potassium (K) within the soil–plant continuum is crucial for elucidating their mechanistic roles in plant growth adaptation. We investigated these processes in the *Suaeda salsa* across a natural salinity gradient in the Yellow River Delta coastal wetlands. Using field surveys, we quantified Na and K enrichment and translocation coefficients among soil, roots, stems, and leaves. The correlation analysis and Random Forest modeling were then employed to identify the key drivers linking these ion dynamics to plant morphological traits (height, density, biomass). Results revealed a pronounced Na compartmentalization, with leaves acting as the primary sink (enrichment coefficient = 5.62), exhibiting values 4.68- and 3.81-fold higher than roots and stems, respectively. In contrast, K enrichment levels remained relatively stable across plant organs (roots: 0.50; stems: 0.57; and leaves: 0.62). Internal Na^+^ loading in stems and leaves positively correlated with leaf Na enrichment. Conversely, high soil Na suppressed both leaf Na enrichment and stem-to-leaf K translocation, while elevated soil K reduced K enrichment in all organs and soil-to-root Na translocation. Critically, plant height was negatively correlated with Na-K enrichment coefficients in all organs. Population density and biomass were specifically linked to stem-related Na dynamics (stem-leaf Na translocation and stem Na enrichment), with K translocation showing no significant relationship. The Random Forest model identified the stem K enrichment coefficient, leaf K content and its enrichment coefficient, and stem Na content as the most influential coefficients governing plant growth (relative importance: 6.37~12.82%). We conclude that the growth adaptation of *S. salsa* in this coastal ecosystem is driven by a synergistic yet organ-specific regulation of Na and K translocation and homeostasis. These findings provide a mechanistic physiological basis for informing ecological restoration strategies of *S. salsa* wetlands and support the sustainable management of estuarine ecosystems.

## 1. Introduction

The coastal wetlands of the Yellow River Delta constitute the youngest and most intact wetland ecosystem within China’s warm temperate zone. As a critical habitat for rare avian species, this region plays an indispensable role in sustaining regional biodiversity [1,2,3]. Positioned at the dynamic interface of land, river, and sea, the delta is highly vulnerable to global climate change and anthropogenic pressures, facing escalating environmental stressors including freshwater scarcity, soil salinization, wetland degradation, and biodiversity loss [4,5,6]. Ecosystem degradation is accompanied by low species diversity and simplified, unstable community structures, which severely constrain regional conservation and sustainable development [7,8]. Research aimed at restoring the ecological resilience of these coastal wetlands is therefore critically needed.

In this context, sodium (Na) and potassium (K) emerge as pivotal cationic regulators of halophyte growth and adaptation [9]. These ions govern fundamental processes including selective uptake, long-distance translocation, tissue-specific partitioning, and cellular ion homeostasis [10,11]. A mechanistic understanding of Na and K dynamics is essential for deciphering the physiological basis of salt tolerance in coastal halophytes and directly informs strategies for the conservation and sustainable management of halophyte resources in vulnerable coastal ecosystems.

*Suaeda salsa* is a widely distributed and ecologically foundational halophyte in the coastal wetlands of the Yellow River Delta [12]. This species employs ion compartmentalization as a key salt-tolerance strategy, whereby absorbed salts are sequestered within specific tissues to minimize cytotoxic effects and enable survival under high salinity [13]. Ecologically, *S. salsa* provides food and habitat for soil fauna and microorganisms, contributes to soil desalination through salt uptake and fixation, and is therefore considered a cornerstone species for coastal wetland restoration and saline-alkali land reclamation [14,15]. It has consequently been prioritized as a primary phytoremediation agent in ecological restoration projects across the region. Existing research on *S. salsa* has largely centered on its general physiological traits, salt-tolerance mechanisms, molecular adaptations, and applied restoration potential [16,17,18]. However, a significant knowledge gap persists regarding the dynamic regulation of Na and K under realistic field salt stress. Crucially, few studies have systematically quantified how the translocation and enrichment dynamics of these ions across the entire soil–plant continuum directly shape the growth performance of *S. salsa* in its natural habitat.

Within plant cells, Na can function as an osmoticum, contributing to water balance maintenance under saline conditions. However, its cytoplasmic concentration must be strictly controlled, as most enzymatic activities are highly sensitive to elevated Na. Excess cytoplasmic Na impairs membrane transporter function and competitively inhibits the uptake of essential nutrients such as K and calcium (Ca^2+^), disrupting ion homeostasis and leading to growth inhibition [19,20]. In contrast, K is a vital macronutrient involved in enzyme activation, protein synthesis, charge balance, and osmotic adjustment. Adequate K nutrition promotes photosynthetic efficiency, sustains turgor pressure, and enhances overall plant stress resilience [21,22]. Ultimately, the content, distribution, and stoichiometry of these key elements dictate plant growth phenotypes and ecological fitness [23].

*S. salsa* exemplifies a salt-accumulating halophyte that employs a dual strategy: it sequesters absorbed Na into vacuoles for osmotic adjustment while efficiently absorbing and retaining K to maintain critical cytosolic Na–K homeostasis [24]. The coordinated translocation and compartmentalization of Na and K are mediated by specific transporters (e.g., High-affinity K^+^ Transporter (HKT) and Inward-Rectifying K^+^ Channel (AKT)), forming the mechanistic basis for its adaptation to saline environments [25,26]. While prior research has documented the impacts of tissue Na and K contents and their ratios on various physiological and ecological traits [27,28,29], the regulatory role of their dynamic translocation processes—from soil uptake through inter-organ partitioning—on whole-plant growth morphology remains poorly understood.

Building upon this framework, the present study employs field investigations in the Yellow River Delta wetlands to elucidate the growth adaptation mechanisms of *S. salsa*, with a specific focus on the translocation and partitioning dynamics of Na and K. Our primary objectives are as follows: (1) to quantify how Na and K pools in different compartments (soil, root, stem, and leaf) influence their respective translocation and enrichment coefficients across the soil–plant continuum; (2) to model the growth response patterns (height, density, and biomass) of *S. salsa* to these ion dynamics using power function analyses; and (3) to identify the key ion-related drivers governing *S. salsa* growth through integrated correlation analysis and Random Forest modeling, thereby revealing its physiological adaptation strategy under variable ion availability and transport efficiency. Prior research indicates that tissue Na:K ratios significantly alter internal ion translocation and compartmentalization in halophytes, and that Na can mitigate K deficiency by enhancing photosynthetic capacity in *S. salsa* [30,31]. We therefore hypothesize that (H1) the absolute contents of Na and K in the system directly regulate their translocation and enrichment patterns, with Na exerting a particularly strong influence on K dynamics. Furthermore, studies show that plant height and biomass decline markedly when Na enrichment exceeds a supra-optimal threshold in coastal wetlands [32,33], leading to our second hypothesis (H2): excessive Na translocation and accumulation negatively impacts the height and biomass of *S. salsa*. Finally, given the recognized strategy of foliar Na sequestration for osmotic adjustment [34,35], we predict (H3) that leaf Na content and its enrichment coefficient are pivotal determinants of *S. salsa* growth performance. By testing these hypotheses, this study moves beyond descriptive correlations to establish a process-driven understanding of growth adaptation. The findings provide a mechanistic scientific basis for leveraging *S. salsa* in the ecological restoration of the Yellow River Delta coastal wetlands.

## 2. Results

### 2.1. Translocation and Enrichment Dynamics of Na and K in the Soil–S. salsa System

**Figure 1:**
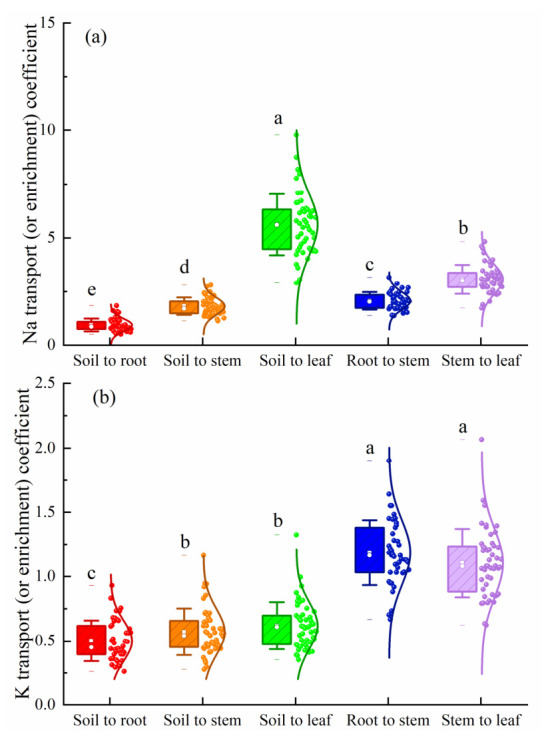
Translocation and enrichment coefficients of Na (**a**) and K (**b**) in the soil–*S. salsa* system of the Yellow River Delta wetlands. Different lowercase letters above bars indicate statistically significant differences among compartments at *p* < 0.05.

We observed significant compartment-specific differences in the translocation and enrichment coefficients of Na and K within the soil–*S. salsa* system (all *p* < 0.05). Notably, Na exhibited a pronounced enrichment in leaves, with a soil-to-leaf Na enrichment coefficient (*ENNa_S-L_*) of 5.62—the highest value recorded across all pathways (Figure 1a). In contrast, the root-to-stem Na translocation coefficient (*TFNa_R-St_*), stem-to-leaf Na translocation coefficient (*TFNa_St-L_*), soil-to-root Na enrichment coefficient (*ENNa_S-R_*), and soil-to-stem Na enrichment coefficient (*ENNa_S-St_*) were substantially lower, ranging from 0.94 to 3.07.

The patterns for K were markedly different. The root-to-stem K translocation coefficient (*TFK_R-St_*) and stem-to-leaf K translocation coefficient (*TFK_St-L_*) were relatively high (1.19 and 1.15, respectively), indicating efficient internal redistribution. However, K enrichment coefficients relative to the soil were low and showed minimal variation among plant organs, with values of 0.50, 0.57, and 0.62 for the soil-to-root K enrichment coefficient (*ENK_S-R_*), soil-to-stem K enrichment coefficient (*ENK_S-St_*), and soil-to-leaf K enrichment coefficient (*ENK_S-L_*), respectively (Figure 1b).

### 2.2. Effects of Internal Na and K Pools on Translocation and Enrichment Dynamics

**Figure 2:**
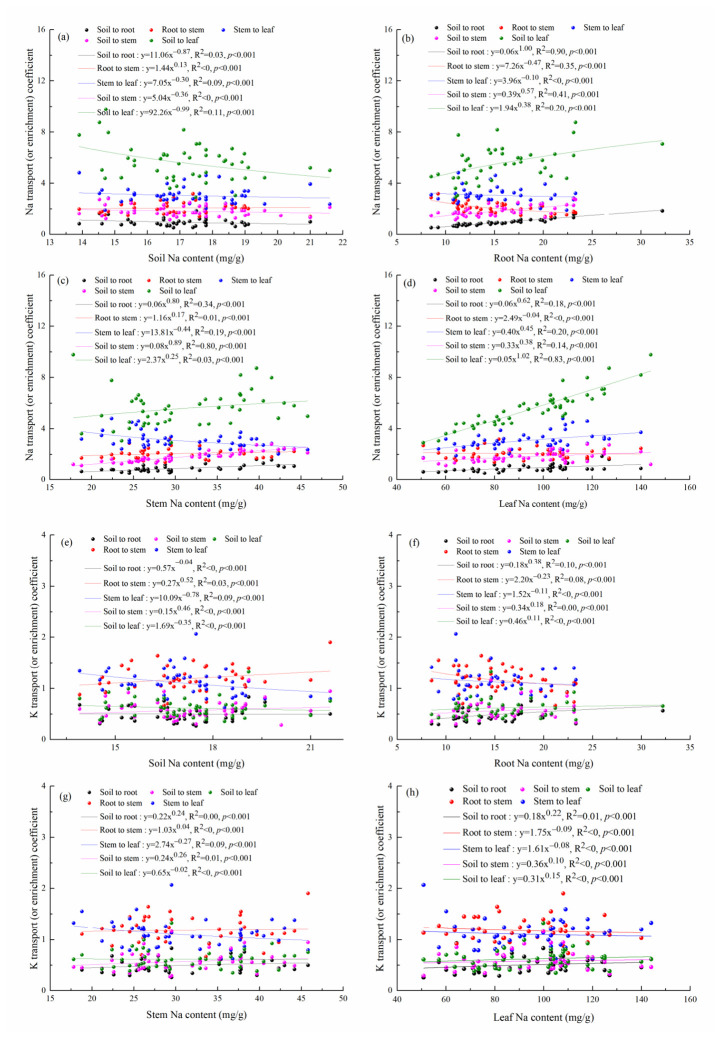
Effects of Na concentrations in different compartments on Na and K translocation and enrichment coefficients within the soil–*S. salsa* system. (**a**) *TF*(*EN*)*Na*–soil Na content; (**b**) *TF*(*EN*)*Na*–root Na content; (**c**) *TF*(*EN*)*Na*–stem Na content; (**d**) *TF*(*EN*)*Na*–leaf Na content; (**e**) *TF*(*EN*)*K*–soil Na content; (**f**) *TF*(*EN*)*K*–root Na content; (**g**) *TF*(*EN*)*K*–stem Na content; (**h**) *TF*(*EN*)*K*–leaf Na content.

The translocation and enrichment coefficients of Na and K responded differentially to variations in ion concentrations across compartments along the soil–root–stem–leaf continuum (Figure 2). Elevated soil Na concentration was associated with a significant decrease in *ENNa_S-R_* and *TFNa_S-L_*, as well as *TFNa_St-L_* and *TFK_St-L_* (exponent < 0). Conversely, the *TFK_R-St_* increased significantly under higher soil Na availability (y = 0.27x^0.52^) (Figure 2a,e). Increased root Na concentration triggered significant changes in most ion translocation and enrichment pathways. The notable exceptions were the *TF_St-L_* for both ions and the *ENNa_S-L_*, which remained stable (Figure 2b,f). When stem Na concentration rose, we observed a pronounced decline specifically in the *TF_St-L_* for Na and K. In contrast, other Na-related translocation/enrichment coefficients, as well as *ENK_S-R_* and *ENK_S-St_*, increased significantly (Figure 2c,g). Finally, higher leaf Na concentration was linked to significant increases in Na enrichment coefficients across all plant compartments, the *ENK_S-R_*, and the *TFNa_St-L_* (Figure 2d,h).

**Figure 3:**
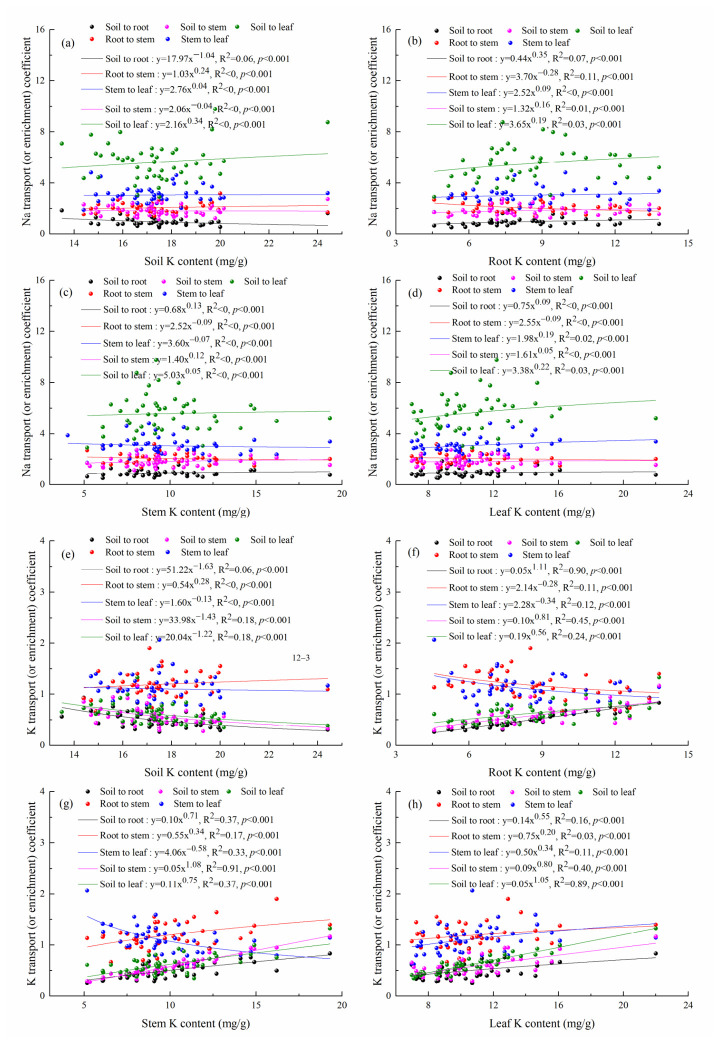
Effects of K concentrations in different compartments on Na and K translocation and enrichment coefficients within the soil–*S. salsa* system. (**a**) *TF*(*EN*)*Na*–soil K content; (**b**) *TF*(*EN*)*Na*–root K content; (**c**) *TF*(*EN*)*Na*–stem K content; (**d**) *TF*(*EN*)*Na*–leaf K content; (**e**) *TF*(*EN*)*K*–soil K content; (**f**) *TF*(*EN*)*K*–root K content; (**g**) *TF*(*EN*)*K*–stem K content; (**h**) *TF*(*EN*)*K*–leaf K content.

Variations in K concentration across different compartments of the soil–*S. salsa* system exerted significant regulatory effects on most Na and K translocation and enrichment coefficients (Figure 3). Elevated soil K concentration was associated with a coordinated downregulation of K enrichment coefficients in all plant organs (*ENK_S-R_*, *ENK_S-St_*, and *ENK_S-L_*)—as well as a significant reduction in the soil-to-root Na translocation coefficient (*ENNa_S-R_*) (exponent < 0) (Figure 3a,e). When stem K concentration rose, it specifically suppressed the *TFK_St-L_*, while simultaneously promoting increases in other K-related translocation and enrichment coefficients (exponent > 0) (Figure 3c,g). Finally, higher leaf K concentration was associated with significant increases in multiple pathways, including the *TFNa_St-L_*, *ENNa_St-L_*, and various K translocation and enrichment coefficients (exponent > 0) (Figure 3d,h).

### 2.3. Growth Response of S. salsa to Na and K Translocation and Enrichment Dynamics

**Figure 4:**
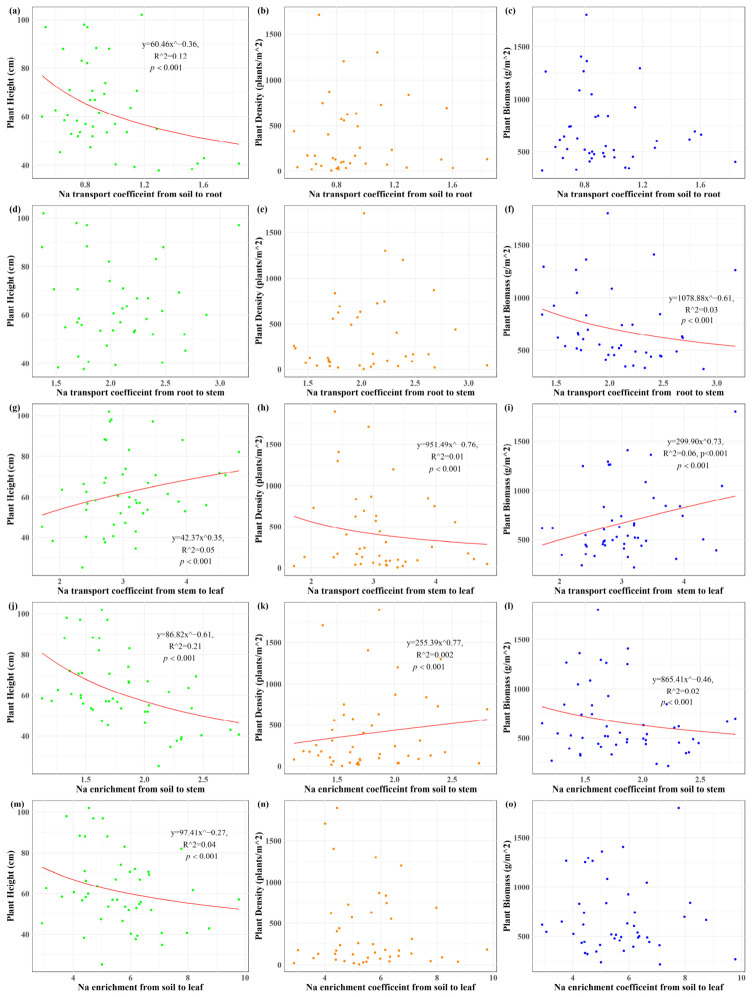
Responses of plant height, population density, and biomass of *S. salsa* to Na translocation and enrichment coefficients. The green, orange, and blue points represent plant height, density and biomass of *S. salsa*, respectively. (**a**–**c**) showed responses of plant height, population density, and biomass of *S. salsa* to *TFNa_S-__R_*; (**d**–**f**) showed responses of plant height, population density, and biomass of *S. salsa* to *TFNa_R-__St_*; (**g**–**i**) showed responses of plant height, population density, and biomass of *S. salsa* to *TFNa_St-__L_*; (**j**–**l**) showed responses of plant height, population density, and biomass of *S. salsa* to *ENNa_S-__St_*; (**m**–**o**) showed responses of plant height, population density, and biomass of *S. salsa* to *ENNa_S-__L_*.

The growth traits of *S. salsa* displayed distinct and significant responses to variations in Na translocation and enrichment factors, whereas K-related factors showed no consistent associations (Figure 4). Plant height exhibited strong correlations with most Na metrics. Height decreased significantly with an increase in sodium enrichment factors in the roots (*ENNa_S-R_*, *ENNa_S-St_*, and *ENNa_S-L_*) (Figure 4a–c; all *p* < 0.001). In contrast, it increased significantly with a higher *TFNa_St-L_* (Figure 4d; *p* < 0.001). The *TFNa_R-St_* was the only Na metric not significantly correlated with plant height (Figure 4e). Plant population density was significantly associated with stem-related Na dynamics. Density decreased significantly as the *TFNa_St-L_* increased (Figure 4h; *p* < 0.001). Conversely, it increased with a higher *ENNa_S-St_*) (Figure 4i; *p* < 0.001). The biomass of *S. salsa* demonstrated pathway-specific responses. It was negatively correlated with *TFNa_R-St_* and with *ENNa_S-St_* (Figure 4l,m; both *p* < 0.001). However, biomass increased significantly with an enhancement in *TFNa_St-L_* (Figure 4n; *p* < 0.001).

**Figure 5:**
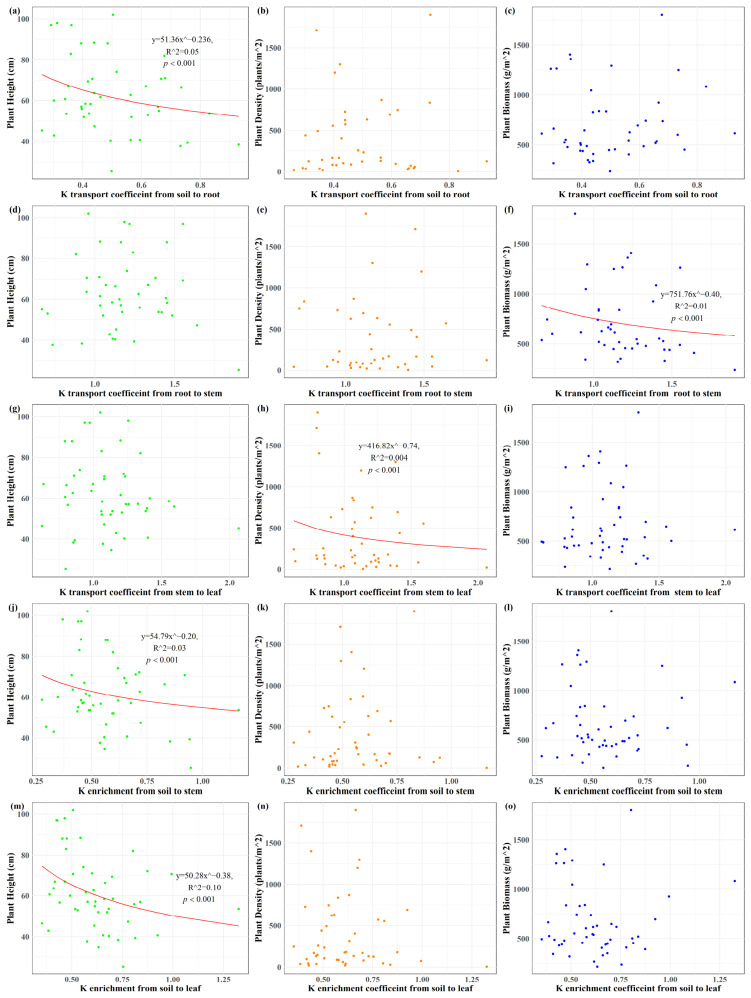
Responses of plant height, population density, and biomass of *S. salsa* to K translocation and enrichment coefficients. The green, orange, and blue points represent plant height, density and biomass of *S. salsa*, respectively. (**a**–**c**) showed responses of plant height, population density, and biomass of *S. salsa* to *TFK_S-__R_*; (**d**–**f**) showed responses of plant height, population density, and biomass of *S. salsa* to *TFK_R-__St_*; (**g**–**i**) showed responses of plant height, population density, and biomass of *S. salsa* to *TFK_St-__L_*; (**j**–**l**) showed responses of plant height, population density, and biomass of *S. salsa* to *ENK_S-__St_*; (**m**–**o**) showed responses of plant height, population density, and biomass of *S. salsa* to *ENK_S-__L_*.

In contrast to the strong and consistent patterns observed for Na, K translocation and enrichment coefficients exhibited more limited and variable associations with the growth of *S. salsa* (Figure 5).

Plant height was the only growth trait consistently influenced by K dynamics. Height showed significant negative correlations with *ENK_S-R_*, *ENK_S-St_*, and *ENK_S-L_* (Figure 5a,d,e; all *p* < 0.001). For plant density and biomass, K-related correlations showed predominantly non-significant relationships (Figure 5f–o). The only exceptions were a significant negative correlation between the *TFK_St-L_* and plant density (Figure 5h; *p* < 0.001) and between the *TFK_R-St_* and biomass (Figure 5l; *p* < 0.001).

### 2.4. Integrated Correlation Network of Ion Concentrations and Translocation Coefficients

**Figure 6:**
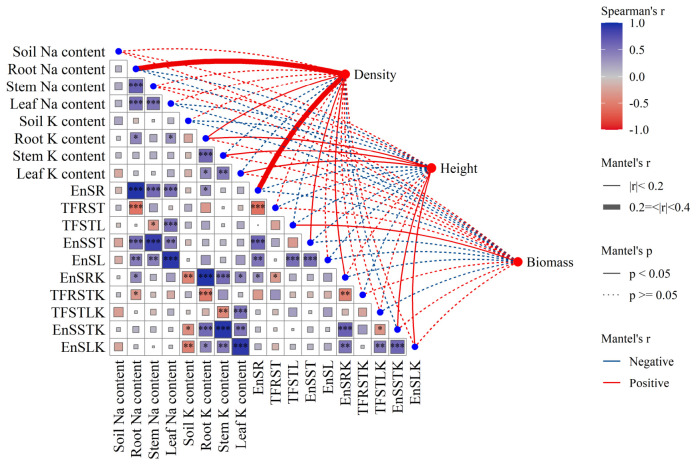
Spearman correlation network and Mantel test analysis integrating plant growth traits with ion dynamics in the soil–*S. salsa* system. S, soil; R, root; St, stem; L, leaf; EN, enrichment factor; TF, translocation factor. Na and K factors are indicated as Na and K subscripts, respectively (e.g., ENNaS-R: soil-to-root Na enrichment coefficient). *, ** and *** in the figure indicate significance *p* < 0.05, *p* < 0.01 and *p* < 0.001, respectively.

A network of correlation analyses and Mantel tests revealed a complex web of interdependencies among ion pools and their translocation dynamics within *S. salsa* (Figure 6). Significant positive correlations were prevalent among Na (or K) concentrations across plant organs, suggesting coordinated ion homeostasis at the whole-plant level. Notably, however, organ-specific coupling between Na and K was observed within individual tissues. Specifically, root Na concentration showed an exclusive positive correlation with root K concentration. A parallel, exclusive positive correlation was found between leaf Na concentration and leaf K concentration. The relationships between ion concentrations and their translocation pathways were also distinct. Na concentrations in roots, stems, and leaves were strongly and positively correlated with their corresponding organ-specific enrichment coefficients (*ENNa_S-R_*, *ENNa_S-St_*, and *ENNa_S-L_*). Furthermore, these Na pools showed positive correlations with the *TFNa_R-St_* and *TFNa_St-L_* pathways. K concentrations across the soil–plant system exhibited significant positive correlations with sodium enrichment factors in all three plant organs (*ENNa_S-R_*, *ENNa_S-St_*, and *ENNa_S-L_*), suggesting a coordinated regulation of Na accumulation and K status. Correlations among the translocation and enrichment coefficients themselves were fewer but structured. Key interconnections included positive correlations between the Na enrichment coefficients (*ENNa_S-R_*, *ENNa_S-St_*, and *ENNa_S-L_*).

Plant growth metrics—density, height, and biomass—demonstrated selective linear associations with specific ion concentrations and translocation coefficients across the soil–*S. salsa* continuum (Figure 6). Population density displayed significant linear correlations with Na-related metrics in roots (Na concentration and *ENNa_S-R_*) and *ENNa_S-St_*, as well as with K metrics in roots (K concentration and *ENK_S-R_*). Plant height showed significant linear relationships exclusively with K pools, being correlated with K concentration in all three organs (root, stem, and leaf) and with the *ENK_S-St_* and *ENK_S-L_*. In contrast, biomass maintained a single, strong positive linear correlation solely with the *TFNa_St-L_*.

**Figure 7:**
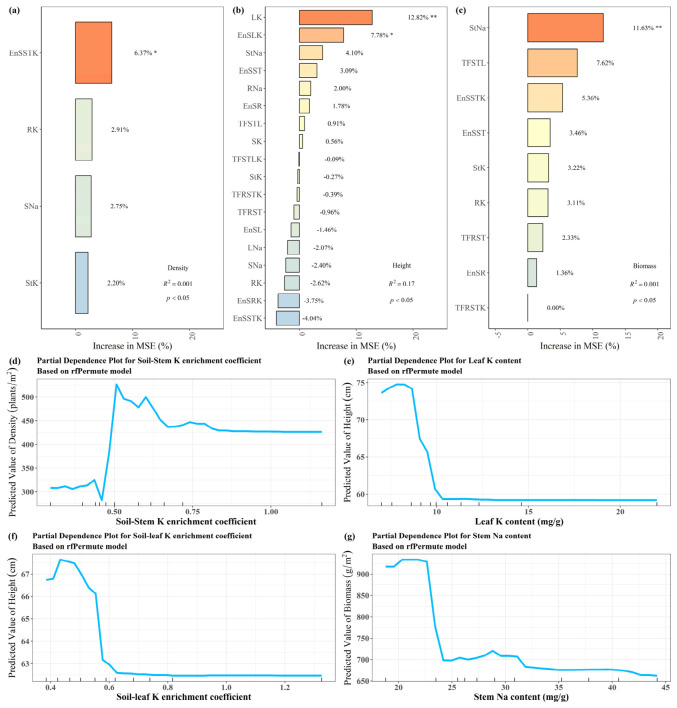
Random Forest model analysis (RFM) of key predictors for *S. salsa* growth traits (density, height, and biomass) from the pool of Na and K concentrations and translocation dynamics. *S*, soil; *R*, root; *St*, stem; *L*, leaf; *EN*, enrichment factor; *TF*, translocation factor. Predictors are denoted by their respective compartment and ion (e.g., *StNa*: stem Na concentration; *ENK_S-St_*: soil-to-stem K enrichment coefficient). * and ** in the figure indicate significance *p* < 0.05, *p* < 0.01, repectively. (**a**–**c**) showed the RFM for density, height and biomass of *S. salsa*, respectively. (**d**–**g**) showed the predicted value of density to *ENK_S-St_*, height to leaf K content, height to *ENK_S-L_*, biomass to stem Na content, respectively.

The Random Forest model analysis identified distinct and key ion-related predictors for each growth metric of *S. salsa* (Figure 7). For population density, the *ENK_S-St_* emerged as the most significant predictor, explaining 6.37% of the variation (%IncMSE) (Figure 7a). Model-predicted density exhibited a non-linear response to an increase in stem K concentration, characterized by an initial rise, followed by a decline and eventual stabilization (Figure 7d). Plant height variation was best explained by two leaf K metrics—leaf K concentration and the *ENK_S-L_*, with relative importance values (%IncMSE) of 12.82% and 7.78%, respectively (Figure 7b). The predicted relationship for both predictors was similar, showing a slight initial increase in height at low predictor values, succeeded by a significant decline and stabilization at low height values (Figure 7e,f). Stem Na concentration was identified as the most important predictor of biomass in the Random Forest model, which was the strongest predictor, with an importance of 11.63% (%IncMSE) (Figure 7c). The predicted biomass response to stem Na concentration was markedly non-linear: a slight initial increase was followed by a sharp decline after a distinct threshold, leading to a fluctuating but overall decreasing trajectory at higher concentrations (Figure 7g).

## 3. Discussion

*Suaeda salsa*, a characteristic halophyte of coastal wetlands, exhibits efficient salt accumulation through both root uptake and foliar absorption [36], demonstrating a pronounced physiological pre-adaptation to Na. Leaves, as the primary sites of gas exchange and transpiration, are known to develop a high capacity for ion accumulation under saline conditions, often serving as terminal sinks for Na sequestration [37,38,39]. Our findings are consistent with this model: the *ENNa_S-L_* was significantly higher than in *ENNa_S-R_* or *ENNa_S-St_* within the soil–*S. salsa* system. This pattern corroborates the established detoxification strategy where roots absorb Na, xylem transport delivers it to the shoot, and leaves ultimately compartmentalize it in vacuoles to mitigate cytosolic toxicity [40]. Furthermore, all measured Na translocation and enrichment coefficients in *S. salsa* were substantially greater than their K counterparts. This disparity can be mechanistically explained by ion competition at key uptake sites. Under elevated external Na, Na directly competes with K for binding sites on shared transport proteins, such as high-affinity K transporters (e.g., the High-Affinity K^+^ Transporter/K^+^ Uptake Permease (HAK/KUP) family) and non-selective cation channels. This competitive inhibition suppresses K influx [41,42], leading to the systematically lower K translocation and enrichment coefficients we observed. These findings support H1, but the regulation is ion-specific and organ-specific rather than uniform across the whole plant.

Variations in soil Na and K availability are a primary driver of ion translocation and partitioning dynamics in coastal halophytes. While Na is cytotoxic to most plants [43], *S. salsa* employs a distinct adaptive strategy: it actively absorbs Na^+^ and chloride (Cl^−^), subsequently sequestering these ions within the vacuoles of photosynthetic mesophyll cells. This compartmentalization serves a dual purpose—it detoxifies the cytosol while simultaneously lowering the vacuolar water potential, thereby facilitating osmotic water uptake from saline soils [24,38]. To maintain ion homeostasis under fluctuating soil ion ratios and environmental conditions, plants dynamically regulate the absorption, long-distance transport, and tissue-specific allocation of Na and K. This continuous physiological adjustment manifests as the observed spatial variation in ion translocation and enrichment factors among roots, stems, and leaves [31,44]. Our results provide clear evidence for such dynamic redistribution. Changes in Na and K pools across the soil–plant system significantly altered K translocation patterns, triggering a reallocation of K among organs. This aligns with the principle that plants can mitigate ionic stress by preferentially accumulating or excluding ions in specific tissues [38]. Specifically, we found that under increasing soil Na concentration, K was preferentially retained in the stem. This was evidenced by an increase in the *TFK_R-St_*, a decrease in the *TFK_St-L_*, and a consequent rise in stem K concentration. Collectively, Na and K translocation and enrichment coefficients are more than mere metrics; they are quantitative signatures of the plant’s integrated response to external ion availability and a reflection of its internal self-regulatory capacity under salt stress. As such, they represent a core physio-ecological strategy underpinning habitat adaptation.

**Figure 8:**
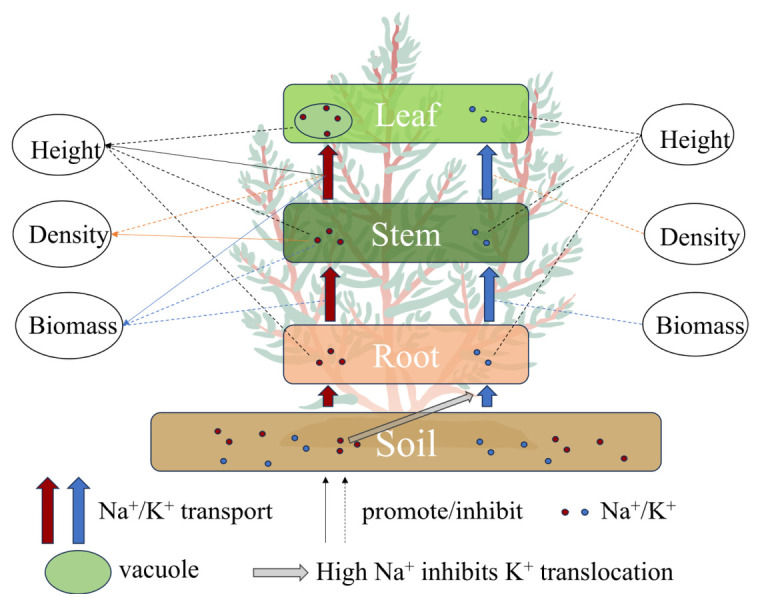
Conceptual model of Na and K translocation, compartmentalization, and growth regulation in *S. salsa*.

The internal translocation and enrichment of Na and K critically shape key growth metrics of *S. salsa*, including height, density, and biomass (Figure 8) [45,46]. However, our analysis reveals that Na and K exert distinct and often contrasting effects. Na dynamics showed clear, organ-specific impacts on plant architecture and population performance. Plant height was negatively correlated with Na enrichment factors in roots, stems, and leaves (Figure 4a,c–e), suggesting that excessive Na accumulation in these tissues directly inhibits vertical elongation. Conversely, the *TFNa_St-L_* was positively associated with height. This pattern indicates a beneficial growth strategy: minimizing Na retention in stems likely facilitates vertical growth, while sequestering Na in succulent leaves, where it can serve as an osmoticum, mitigates its toxic effects [24,38]. Population density and biomass were most sensitive to Na dynamics within the stem. Moderate stem Na accumulation was positively linked to both density and biomass. This likely reflects the dual role of Na in halophytes: at controlled levels, it contributes to osmotic adjustment, lowering the cellular water potential to facilitate turgor maintenance and growth in saline soils [47]. Therefore, H2 and H3 were partially supported: excessive Na enrichment was associated with reduced growth. The influence of K on plant density and biomass was more limited and complex. Only specific translocation pathways—the *TFK_St-L_* and *TFK_R-St_*—showed significant correlations. This suggests that K’s role in shaping these population-level metrics is not simply a function of tissue concentration but is finely regulated by its internal routing. The overall impact of K is likely modulated by a suite of interacting factors, including soil chemistry, ion antagonisms, and microenvironmental conditions [18,48], pointing to a multifaceted regulatory mechanism that warrants further mechanistic dissection.

The Yellow River Delta wetlands represent a classic coastal salinized ecosystem where soil salinity is a primary environmental filter, critically shaping community assembly and challenging restoration efforts. *Suaeda salsa*, a pioneer halophyte in this system, has evolved specific physiological strategies for the acquisition, translocation, and partitioning of Na and K. A mechanistic understanding of these strategies provides an indispensable theoretical foundation for the science-based restoration of such degraded wetlands. Practically, the insights derived from quantifying Na and K translocation and enrichment factors can inform targeted management interventions. For instance, the human-assisted regulation of the soil Na:K ratio could be optimized to favor K uptake efficiency in *S. salsa*, thereby mitigating the competitive inhibition exerted by Na and enhancing plant establishment and growth during restoration. While this study delineates the characteristics and ecological significance of internal Na and K dynamics in the soil–*S. salsa* system, it is important to acknowledge its scope. Our investigation provides a snapshot from a specific growing season and thus does not capture potential seasonal variations in ion translocation patterns. Furthermore, we focused on the pivotal roles of Na and K; in natural saline environments, other ions such as chloride (Cl^−^) and calcium (Ca^2+^) are integral to osmotic adjustment and ionic homeostasis. The underlying molecular regulatory networks governing Na^+^ and K^+^ transport also remain to be fully elucidated. Future research should therefore expand along several fronts. Investigations across multiple seasons are needed to resolve the temporal dynamics of ion translocation. A more comprehensive ionomic approach, coupled with molecular and genetic techniques, will be essential to unravel the synergistic interactions between multiple ions and their transporters. By integrating these scales—from seasonal field patterns to molecular mechanisms—we can build a robust and predictive framework to guide the effective ecological restoration of salinized wetlands in the Yellow River Delta and analogous ecosystems globally.

## 4. Materials and Methods

### 4.1. Study Area

**Figure 9:**
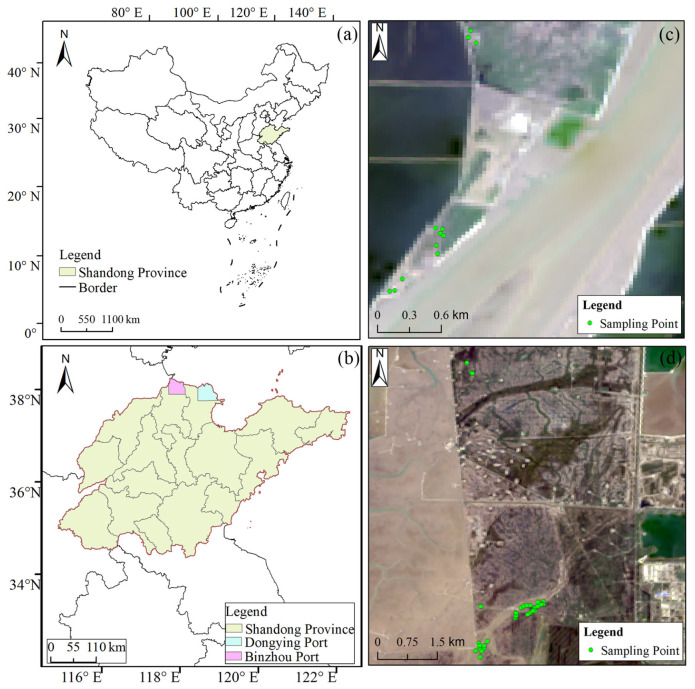
Geographic location of the study area and spatial distribution of *S. salsa* sampling sites in the coastal wetlands of the Yellow River Delta. (**a**) China; (**b**) Shandong Province; (**c**) Binzhou Port; and (**d**) Dongying Port.

The study was conducted in the coastal wetlands of the Yellow River Delta (36°55′ N–38°16′ N, 117°31′ E–119°18′ E), located in northeastern Shandong Province, China (Figure 9). The region experiences a warm temperate continental monsoon climate, marked by distinct seasonal fluctuations: hot and humid summers contrast with cold and dry winters [49]. The mean annual temperature is 13.5 °C. Annual precipitation, averaging approximately 600 mm, exhibits pronounced inter-annual variability. This is coupled with a high potential evapotranspiration rate (~1175 mm year^−1^), creating a significant water deficit that exacerbates soil salinization [50]. The wetland soils are primarily alluvial in origin and are classified as saline, saline-alkali, and coastal saline soils. The soluble salt content of the tested soil was 7.49 g/kg [51,52]. The area is dominated by salt-tolerant herbaceous vegetation, with a regional mean NDVI value below 0.20 [53,54]. In recent decades, the synergistic pressures of global climate change and intensive local industrial and agricultural development have subjected these coastal wetlands to increasingly complex environmental stressors. These compounding pressures have accelerated soil salinization and significantly heightened the vulnerability of the entire wetland ecosystem [45,55,56]. *Suaeda salsa* is a widely distributed and highly stress-tolerant halophyte across these degraded wetlands. Its pronounced resilience to salinity and drought makes it a keystone species for phytoremediation and a critical biological agent in ongoing coastal wetland restoration projects, playing an indispensable role in the functional recovery of these threatened ecosystems.

### 4.2. Collection and Analysis of Plant and Soil Samples

The field investigation was conducted during the peak growth season (July–August) of 2024, targeting natural populations of *S. salsa* across the coastal wetlands of the Yellow River Delta. We established a total of 51 sampling quadrats (50 cm × 50 cm) distributed across the study region, comprising 12 quadrats near Binzhou Port and 39 near Dongying Port, to capture spatial environmental heterogeneity. The two sampling regions represent typical coastal wetland habitats with naturally developed *S. salsa* populations. Within each region, quadrats were established in relatively homogeneous *S. salsa* patches while avoiding severely disturbed areas, roadsides, and artificially managed sites. The precise geographical coordinates of each quadrat were recorded using a handheld GPS receiver. Within each quadrat, we measured the height of three randomly selected *S. salsa* individuals in the sampling quadrats. The mean of these three measurements was calculated to represent the average plant height. The population density is obtained by manually counting within the sample plot. Entire *S. salsa* plants (including roots) and associated soil samples (0–20 cm depth) were systematically collected from each quadrat. All samples were immediately placed in pre-labeled sampling bags and transported on ice to the Shandong Key Laboratory of Eco-Environmental Science for the Yellow River Delta for subsequent processing.

Upon arrival at the laboratory, soil samples were air-dried at room temperature and then sieved (<2 mm) for analysis. Plant samples were carefully cleaned to remove adhered soil and debris, and they were then separated into three organ types: roots, stems, and leaves. Each organ fraction was placed in a labeled paper bag. To halt enzymatic activity, samples were first oven-dried at 120 °C for 2–3 h, and they were then dried to constant weight at 75 °C for biomass determination. The dried plant materials and prepared soils were separately ground to a fine powder using a ball mill for subsequent chemical analysis. Total Na and K concentrations in plant tissues and soils were determined by flame photometry. Prior to analysis, powdered samples were digested in a hot acid mixture (e.g., HNO_3_-HClO_4_) to remove organic and mineral matrices. The resulting digests were filtered, diluted to a known volume with deionized water, and then analyzed against standard curves. Translocation and enrichment coefficients for Na and K between soil and plant compartments were calculated based on their respective measured concentrations (Equation (1)) [57].

(1) $${TF}_{a\text{-}b}(\mathrm{Or}\;{EN}_{a\text{-}b})\;=\frac{C_{b}}{C_{a}}$$

Here, *TF_a-b_* (or *EN_a-b_*) represents the translocation coefficient (or enrichment coefficient) of a target element from compartment “*a*” to compartment “*b*” within the soil–*S. salsa* system. It is calculated as the ratio of the element’s concentration in the recipient compartment (“*b*”) to that in the source compartment (“*b*”). *C_a_* and *C_b_* denote the element concentrations (e.g., for Na or K) in compartment “*a*” and “*b*”, respectively. The system compartments are defined as follows: soil (*S*), root (*R*), stem (*St*), and leaf (*L*). Specifically, translocation coefficients (*TF*) quantify element movement between plant organs, calculated for the root-to-stem (*TF_R-St_*) and stem-to-leaf (*TF_St-L_*) pathways. Enrichment coefficients (*EN*) quantify element accumulation relative to the soil source, calculated for the soil-to-root (*EN_S-R_*), soil-to-stem (*EN_S-St_*), and soil-to-leaf (*EN_S-L_*) pathways.

### 4.3. Statistical Analyses

All data processing, statistical analyses, and visualizations were performed using Microsoft Office Excel 2019, SPSS 26.0, Origin 2024, and R (version 4.3.0). Prior to analysis, the distributions of all Na and K translocation and enrichment coefficients were assessed for normality using the Shapiro–Wilk test. Data that violated the assumption of normality were either lg- or square-root-transformed. For datasets where transformation did not achieve normality, permutation tests were performed using the ImPerm package (version 2.1.4) in R to ensure the robustness of subsequent non-parametric analyses. One-way analysis of variance (ANOVA) was applied to evaluate significant differences in Na and K translocation and enrichment coefficients across different system compartments (soil, root, stem, and leaf). Where ANOVA indicated significant differences (*p* < 0.05), the Duncan test was employed for pairwise comparisons. To quantify the allometric scaling relationships, we employed power function models (Equation (2)) [58] of the following form:

(2) $$y\;=\;ax^{b}$$

In this model, y represents the dependent variable (e.g., a translocation/enrichment coefficient, or a growth trait such as plant height, density, or biomass), *x* represents the independent variable (e.g., Na or K concentration, or *a* corresponding translocation/enrichment coefficient), and *b* denotes fitted scaling and exponent coefficients, respectively. Finally, to identify the key drivers of *S. salsa* growth, we conducted an integrated analysis. Pairwise correlations between all growth characteristics and ion-related metrics (concentrations and translocation/enrichment coefficients) were examined using Pearson’s or Spearman’s correlation coefficients based on data distribution. Subsequently, a Random Forest regression model was implemented using the randomForest package (version 4.7-1.2) in R, with growth traits as response variables and the full suite of ion metrics as predictors. The relative importance of each predictor was quantified based on the percentage increase in the model’s mean squared error (%IncMSE) when that variable was permuted. All correlation and variable importance analyses were aided by the linkET package (version 0.0.7.4) for visualization and synthesis.

## 5. Conclusions

This study demonstrates that the internal translocation and partitioning of Na and K are fundamental drivers of growth adaptation in *Suaeda salsa* within the coastal wetland ecosystem of the Yellow River Delta. Our findings reveal that specific components of this ion transport network exert distinct and significant control over plant growth characteristics. A key feature of this adaptive strategy is the pronounced capacity of leaves to accumulate Na, with the leaf Na enrichment factor (*ENNa_S-L_*) increasing concomitantly with Na pools in roots, stems, and leaves. Conversely, the dynamics of K were regulated by its availability in both soil and plant tissues. The integrated analysis reveals that Na and K pools, along with their translocation and enrichment factors across the soil–root–stem–leaf continuum, directly and indirectly shape plant performance. Crucially, our results identify organ-specific regulatory nodes: (1) population density was primarily governed by the stem K enrichment factor (*ENK_S-St_*); (2) plant height was negatively correlated with leaf K concentration and its enrichment factor (*ENK_S-L_*); and (3) biomass was strongly constrained by stem Na concentration. In conclusion, the growth of *S. salsa* is not a simple function of bulk ion content but is orchestrated by the synergistic yet compartment-specific regulation of Na and K translocation and homeostasis. This work significantly advances our mechanistic understanding of ion dynamics in the halophyte soil–plant system and provides a vital physiological framework to inform the ecological restoration of *S. salsa* in the threatened coastal wetlands of the Yellow River Delta.

## Data Availability

The data presented in this study are available upon request from the corresponding authors.

## Abbreviations

*TF*	translocation coefficients
*EN*	enrichment coefficients
Na	sodium
K	potassium
*S*	soil
*R*	root
*St*	stem
*L*	leaf
